# Effect of pomegranate juice pre-treatment on the transport of carbamazepine across rat intestine

**Published:** 2010

**Authors:** D. Adukondalu, Y. Shravan Kumar, Y. Vamshi Vishnu, R. Shiva Kumar, Y. Madhusudan Rao

**Affiliations:** Centre for Biopharmaceutics and Pharmacokinetics, University College of Pharmaceutical Sciences, Kakatiya University, India

**Keywords:** Everted sac, Non-everted sac, Induction, CYP3A4

## Abstract

**Background and the purpose of the study:**

Many drug substances along with a variety of naturally occurring dietary or herbal components interact with the CYP enzyme system. The present study was aimed to investigate the effect of pomegranate juice pre-treatment on the transport of carbamazepine across the rat intestine.

**Methods:**

The transport of carbamazepine across different parts of rat intestine was studied by everted and non-everted sac methods. The control and pomegranate juice (10 ml Kg^−1^ for 7 days) pre-treated rats were sacrificed and isolated the intestine. The sacs of intestine were prepared, treated with carbamazepine solution and then placed in dulbeccos buffer. Samples were collected periodically and the drug content was estimated using HPLC.

**Results and conclusion:**

The results show that there was a significant (p<0.05) difference in the transport of carbamazepine from the intestinal sacs of pretreated with pomegranate juice and control. It seems that pomegranate juice might have induced CYP3A4 enzymes and hence drug is extensively metabolized

## INTRODUCTION

Drug substances along with dietary or herbal components may interact with CYP enzyme system and induce or inhibit one or more CYP iso-forms which result in increase or decrease of the plasma drug concentration. Plant products with a number of ingredients have diverse chemical nature and increase in their uses may raise the possibility for drug-drug and/or drug-food interactions which may alters drug bioavailability through its effect on absorption, distribution and elimination.

The CYP3A families of enzymes constitute the most predominant phase I drug metabolizing enzymes estimated to metabolize 50-70% of currently administered drugs. CYP3A4 is the most abundant form (1, 2) present primarily in the hepatocytes and enterocytes (3).

Pomegranate (*Punica granatum*) fruits are globally consumed in fresh, processed forms such as juice, jam, wine and oil and in extract supplements. They contain diverse range of phytochemicals including polyphenols like punicalagin (PA), ellagic acid (EA), gallotannins, anthocyanins and other flavonoids (4). PA is the most abundant of these polyphenols, and EA has shown anti-carcinogenic properties, through induction of cell cycle arrest as well as apoptosis and inhibition of tumor formation and growth in animals.

Pomegranate juice consumption has shown potent antioxidant activity, resulting in beneficial health effects such as inhibition of low density lipoprotein oxidation and decrease in cardiovascular diseases (5). High pomegranate consumption may increase the possibility of interaction with drugs. Hence it is important to assess this effect as there are few reports on CYP3A-mediated drugs interaction.

Carbamazepine is an anticonvulsant which is used in the treatment of partial seizures, tonic-clonic seizures and trigeminal neuralgia. Simultaneous consumption of grapefruit juice with a number of therapeutic agents that are subject to first pass intestinal/hepatic metabolism have resulted in higher plasma levels with subsequent adverse effects and inhibition of intestinal CYP3A4 (6, 7).

The present study was aimed to investigate the effect of pomegranate juice pre-treatment on the transport of carbamazepine with involvement of CYP3A4.

## MARERIAL AND METHODS

### Materials

Carbamazepine and sodium butyrate were gifts from Dr. Reddy's Lab Ltd.(Hyderabad, India), Dulbeccos phosphate buffer pH of 7.4 (Hi Media Ltd Mumbai, India), Methanol, Acetonitrile (E. Merck Ltd Mumbai, India) and all chemicals used in this study were of AR grade.

#### Preparation of pomegranate juice

Pomegranate fruits were cut into pieces, removed the rind, separated the seeds, grounded in a mixer (Remi, Mumbai, India) and then filtered to get clear juice. The juice used in this study was collected from the pomegranate fruits of the same plant.

#### Experimental animals

Male Wistar rats weighing about 200*±*25 g were selected and the study was conducted according to the protocol approved by animal ethics committee, Kakatiya University, India.

### Methods

#### In vitro transport study

The transport of carbamazepine across rat intestine (duodenum, jejunum, ileum and colon) was studied by using in vitro everted (15) and non-everted sac methods (8). The rats were treated separately with pomegranate juice (10 ml.Kg-1) and sodium butyrate (0.5 mg.Kg−1) in groups of 3 for 7 days sacrificed, isolated the intestinal segments isolated and then the sacs were prepared. The drug solution was placed in sac and kept in dulbeccos buffer. Samples were collected at pre-set time points by replacing with fresh buffer and their drug contents were estimated using validated HPLC method. Control experiments were also performed.

#### Precipitation method

Methanol (100 µl) was added to intestinal sac samples (200 µl) vortexed on cyclomixer for 2 min, centrifuged at 2500 rpm for 15 min using Biofuge Fresco Centrifuge (Heraeus, Germany) and supernatant was separated. Twenty microlitres of the supernatant was taken into Hamilton syringe and injected into HPLC column.

#### HPLC Analysis

Shimadzu HPLC system equipped with a LC-10AT pump and SPD 10 AT UV visible detector and RP C18 column (120 mm x 4.6 mm ID, particle size 5 µ) was used for the analysis of samples. The mobile phase was a mixture of methanol and water (50:50). The flow rate was 1 ml.min−1and the detection was carried out at 270 nm. The calibration curve was plotted in the range of 3.125 to 50 µg.ml−1. A linear relationship was observed between the concentration and the peak area of carbamazepine with a correlation coefficient (r2=0.999).

The required studies were carried out to estimate the precision and accuracy of the HPLC method of analysis of carbamazepine. The limit of detection was 0.25 µg.ml−1. The average recovery of the drug was 95.75%. The intra day R.S.D (%) was less than 7. 29% and the inter day R.S.D (%) was less than 7. 64% (n=5).

#### Statistical analyses

The in vitro results were compared by student t-test using Sigma Stat Software (Jandel Scientific Sigma stat version 1, 1992-94). A value of P<0.05 was considered to be statistically significant.

## RESULTS AND DISCUSION

In the present study, the amount of transport of carbamazepine from non-everted sac (mucosal to serosal surface) and everted sac (serosal to mucosal surface) were determined in different regions of the rat intestine of the control, pomegranate and sodium butyrate treated groups (Table 1). Results of this study reveals that the transport of carbamazepine in non-everted and everted sac methods was reduced by the treatment of pomegranate juice, in comparison to the control and sodium butyrate treated groups.

**Table 1:** Cumulative amount (µg) of carbamazepine (Mean±S.D) transported from different parts of albino wistar rat small intestine (n=3) after 120 min.

Part of Intestine	Control group		Pomegranate treated group		Sodium.butyrate treated group	
		
	Non-everted (µg)	Everted (µg)	Non-everted (µg)	Everted (µg)	Non-everted (µg)	Everted (µg)
**Duodenum**	12.4±0.14	13.1±0.2	7.07± 1.0	12.29±0 .7	11.9±1.0 †	15.5±0.3
**Jejunum**	13.3±0.66	15.2±0.2	8.96±0 .32	11.87±0.3	13.4±0 .4†	14.3±0.3
**Ileum**	9.97±0.77	17.1±0.3	7.97±0.05	14.82 ±0.4	10.3±0.8	16.8±0.8†
**Colon**	9.57±0.48	14.0±0.2	6.85±0.7	9.89±0.1	9.7±1.2†	14.5±0.3†

Note: † groups with no significant P value

The percentage of the change in drug transported was high in all parts of intestine treated with pomegranate juice and very low in the case of sodium butyrate treated group.

A growing number of studies have documented interaction of grape juice with drugs that are metabolized by the cytochrome P450 3A subfamily (CYP3A), in particular by the CYP3A4 and CYP3A5 enzymes.

Carbamazepine is mainly metabolized by CYP3A4 to active metabolite carbamazepine- 10, 11-epoxide. Some reports have shown that carbamazepine regulates intestinal p-glycoprotein and multi-drug resistance protein and influences disposition of talinolol in humans (9).

On the basis of a linear relationship between in vitro everted sac and the in vivo studies (10) it has been suggested that in vivo P-gp related drug-drug interactions can be predicted by in vitro everted sac studies. These workers also suggested that drug-drug interactions related to P-gp mediated transport in human intestine could be predicted by in vivo (exsorption across rat ileum) or in vitro (everted rat intestine) transport study using rat ileum which are comparable with the transport studies in Caco-2 cell monolayers. The non- everted sac model was originally used to evaluate drug transport mechanisms (11). Evered gut sac method also has been used to assess the role of P-glycoprotein on the intestinal secretion of the ivermectin (12).

In the earlier studies it was reported that pomegranate juice influenced the intestinal transport of buspirone in rats (13). It have been reported that the component (s) of pomegranate inhibit the CYP3A- mediated metabolism of carbamazepine (14) by a manner similar to that of grapefruit.

Results of this in vitro study revealed that carbamazepine transport across the small intestine is much affected by pomegranate juice pre-treatment. In this study, the Mean±S.D. cumulative absorption and exsorption concentrations of carbamazepine decreased after pre-treatment with pomegranate juice. In sodium butyrate treated group significant difference was not observed in normal and everted sac studies. This observation indicated the role of CYP3A4 on carbamazepine metabolism. The study revealed that pre-treatment with pomegranate juice acts as CYP3A4 inducer.

**Figure 1:**
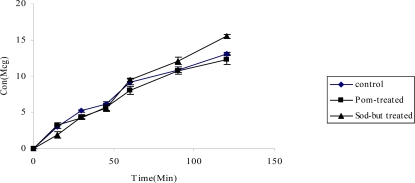
Cumulative transport pattern of carbamazepine in duodenum everted sac in Wistar rats.

**Figure 2:**
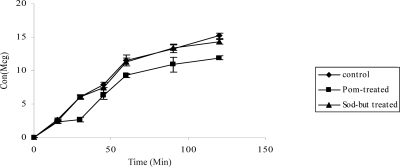
Cumulative transport pattern of carbamazepine in jejunum everted sac in Wister rats.

**Figure 3:**
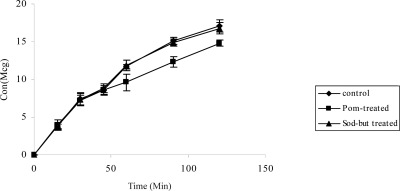
Cumulative transport pattern of carbamazepine in ileum everted sac in wister rats.

**Figure 4:**
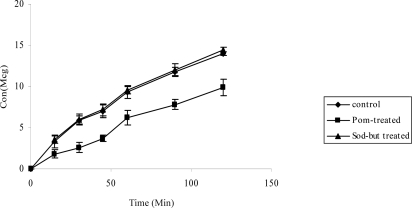
Cumulative transport pattern of carbamazepine in colon everted sac in Wistar rats.

**Figure 5:**
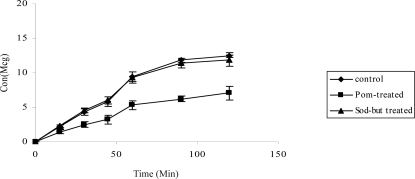
Cumulative transport pattern of carbamazepine in duodenum non-everted sac in Wistar rats.

**Figure 6:**
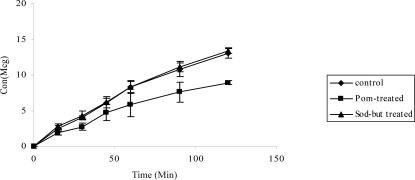
Cumulative transport pattern of carbamazepine in jejunum non-everted sac in Wistar rats.

**Figure 7:**
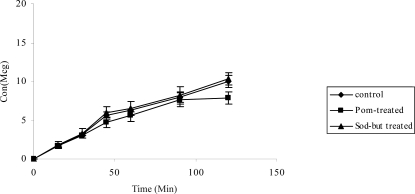
Cumulative transport pattern of carbamazepine in ileum non-everted sac in Wistar rats.

**Figure 8:**
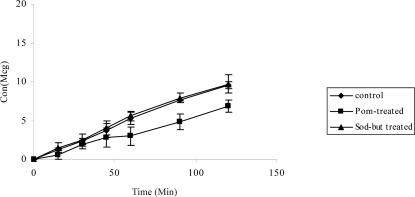
Cumulative transport pattern of carbamazepine in colon non-everted sac in Wistar rats.

**Table 2:** The percentage of changes in cumulative amount of carbamazepine transport in pomegranate juice and sodium butyrate treated groups.

Part of intestine	Pomegranate treated		Sodium butyrate treatedNon	
	
	Non-everted (%)	Everted (%)	Non-everted (%)	Everted (%)
**Duodenum**	42.9	6	4	18
**Jejunum**	31.6	22	2	5
**Ileum**	20	13	7	1.7
**Colon**	28.4	29.3	2	3.5

Pomegranate treated Sodium butyrate treated

## CONCLUTION

The pomegranate juice pre-treatment decreased intestinal permeation of carbamazepine which may be due to induction of CYP3A4 enzyme. Though there are reports on inhibition of carbamazepine metabolism by short pre-treatment with pomegranate juice (14), pre-treatment of rats for longer period (i.e, 7 days) decreased intestinal permeation of carbamazepine probably due to induction of CYP3A4 enzyme. Further studies are recommended to prove their effects in human beings or animals.
